# Cultural variations in perceptions and reactions to social norm transgressions: a comparative study

**DOI:** 10.3389/fpsyg.2023.1243955

**Published:** 2023-09-20

**Authors:** Xing J. Chen-Xia, Verónica Betancor, Laura Rodríguez-Gómez, Armando Rodríguez-Pérez

**Affiliations:** Department of Cognitive, Social and Organizational Psychology, Faculty of Psychology, University of La Laguna, Santa Cruz de Tenerife, Spain

**Keywords:** social norms, cultural differences, collectivism, moral judgment, dehumanization

## Abstract

**Introduction:**

Humans are similar but behave differently, and one main reason is the culture in which they are born and raised. The purpose of this research is to examine how the perception and reaction to those who transgress social norms may vary based on the individualism/collectivism of their culture.

**Methods:**

A study (*N* = 398) conducted in the United Kingdom, Spain, and China showed differences in the perception and reaction to incivilities based on individualism/collectivism.

**Results:**

People from highly collective countries (China) perceive uncivil transgressors as immoral and enact more social control over them than people from highly individualistic countries (U.K.). They also experience more discomfort when facing uncivil transgressors, and this discomfort mediates the increasing immorality perceived on the agents of incivilities in contrast with people from less collective countries.

**Discussion:**

Our findings provide insights into how cultural factors shape individuals’ perceptions of social norm violations and emphasize the importance of considering cultural differences when addressing incivility.

## 1. Introduction

Social norms, which are “behaviors of group members that act as implicit rules, considered to be both descriptive of what group members are and prescriptive (injunctive) of how they should be” (Fiske, 2004, p. 484), are influenced by culture and play a crucial role in shaping cultural differences. Social science has come a long way in expanding its research into other countries; however, most cross-cultural studies are still primarily carried out in Western societies where most studies are WEIRD (conducted with participants who are white, educated, industrialist, rich, and democratic). Western concepts and ideas continue to dominate theories, frameworks, research plans, data collection strategies, analyses, and interpretations in research on cross-cultural values, beliefs, and morality, despite some expansion to include data from previously understudied nations and regions (Goodwin et al., 2020). Traditionally, studies on civility and morality have concentrated on Western societies, but it is now more crucial than ever to consider Eastern cultures that place a greater emphasis on collectivism, interpersonal harmony, and respect for authority (Schwartz, 1994). Therefore, it is necessary to consider cultural differences in values and norms that may exist between Western and Eastern cultures when studying civility and morality.

Non-Western cultures, whose moral practices and beliefs might be dissimilar from those in the West, have largely been ignored in this field. It is necessary to note that Western moral tradition focuses more on deontological ethics, where moral obligations are viewed as fact-like requirements of behavior that can be generalized to other situations (Angle and Slote, 2013). Eastern countries, such as China, are strongly influenced by Confucianism, a form of virtue ethics that emphasizes politeness and everyday courtesy to train character (Hursthouse, 2013; Sarkissian, 2014). In this sense, the cross-cultural study by Buchtel et al. (2015) found that though immorality is usually seen as a universal concept, the behaviors that are considered immoral vary greatly between countries, especially between Western countries where harmful behaviors are considered immoral and Eastern countries where uncivilized behaviors are considered immoral. That means, though individualistic cultures, like the United Kingdom, may place a higher priority on individual liberties and rights, collectivist cultures, like China, may place a higher emphasis on harmony, group cohesion, and the social norms that maintain them.

The degree to which a social norm is respected and followed can influence the impact of incivilities, which influenced our interest in the cultural dimension of individualism and collectivism. Hofstede (2001)’s definition of individualism and collectivism described individualism as a cultural dimension that values individual autonomy and independence, where individuals prioritize their own interests over group interests. On the other hand, collectivism emphasizes the importance of group harmony, interdependence, and loyalty, where individuals prioritize the interests of the group over their own interests. Countries differ in their endorsement of collectivistic or individualistic values, and this can influence people’s reactions to incivilities. As seen in a study in eight countries by Brauer and Chaurand (2010), they observed that the more individualistic a country was, the less they enact social control over uncivil behaviors. However, all eight countries were Western, and we claim Eastern nations must be taken into account to fully comprehend the complexities of civility and morality across various cultures. The present study aims to explore the differences in the reaction to incivilities between Eastern and Western countries that differ in the cultural dimension of individualism/collectivism. Specifically, we selected the highly individualistic United Kingdom, Spain, which falls in between individualism/collectivism, and the highly collectivistic China, with scores of 89, 51, and 20 in individualism, respectively (Hofstede, 2001).

Individuals in collectivistic cultures tend to have a more interdependent definition of self and feel more interconnected with others (Markus and Kitayama, 1991; Triandis, 2001), which may make them feel more personally implicated when witnessing a norm transgression. Furthermore, collectivistic cultures place a greater emphasis on their neighborhood and view uncivil behaviors that harm the neighborhood as more problematic. Therefore, individuals from collectivistic cultures would be more likely to react to social norm transgressions by exerting social control over the transgressor compared to those from individualistic cultures (Triandis, 1995). Additionally, when a social norm is transgressed, it implies a lack of regard and a violation of mutual respect that can determine how negative people feel when mistreated (Cialdini et al., 1990; Reno et al., 1993; Bendor and Swistak, 2001). Being the target of a norm transgression is unpleasant and has been linked to negative feelings (Ekman, 2004; Porath and Pearson, 2012, 2013). The more a person’s actions violate these norms, the more unpleasant people’s experience of the incident (Costa-Lopes et al., 2013). Facing incivilities create discomfort (Moon et al., 2018) and even moral outrage (Moisuc et al., 2018). Moreover, this experience can also be influenced by the culture; specifically, Moon et al. (2018) found that the degree of power distance in different cultures influences the victim’s acceptance of incivilities, particularly when the behaviors are exhibited by individuals in powerful positions within their organization. However, the acceptability of incivilities is not only influenced by the cultural dimension of power distance but also by the cultural dimension of tightness/looseness, that is, the strength and tolerance of social norms (Gelfand et al., 2011). Thus, people from countries with high power distance find incivilities more acceptable, but they also feel great discomfort, which may be influenced by their cultural tightness regarding the importance of norms (Moon and Sánchez-Rodríguez, 2021).

Even more, the impact of social norm transgressions is not limited to those who experience them but can also influence how transgressors are perceived and evaluated. Those who engage in uncivil behaviors may face not only social disapproval but also dehumanization and moral condemnation (Kelman, 1973; Brauer and Chaurand, 2010; Bastian et al., 2013; Buchtel et al., 2015). Social control reactions can differ and be influenced by personal implications, and people may have different perceptions and reactions to norm violations and transgressors (Chekroun and Brauer, 2002; Brauer and Chekroun, 2005; Nugier et al., 2009; Moisuc et al., 2018). Dehumanization can impact the extent to which others view the transgressor as deserving of moral concern, as well as the level of blame assigned to them (Bastian et al., 2011). However, not only is there a gap in the literature of dehumanization enacted by people that are not from WEIRD countries (Ceci et al., 2010; Henrich et al., 2010), there are also differences in how this dehumanization happens. Chen-Xia et al. (2022) observed that gender plays a significant role in the dehumanization of uncivil agents, the lack of stereotypically feminine traits leads to being seen as less human than others. Additionally, Buchtel et al. (2015) observed that transgressing social norms may lead to moral condemnation that can be influenced by the Eastern or Western culture of the ones involved. However, they only observed the behaviors. In this sense, we also seek to study differences regarding the transgressor of social norms. With this in mind, this study aims to examine the perception and reaction to uncivil transgressors based on the cultural differences of individualism/collectivism.

### 1.1. The present study

The purpose of this research is to examine the relationship between culture and incivility. Specifically, we intend to study how the reaction to those who transgress social norms may vary based on their individualistic/collectivistic cultures.

The perception of social norm transgressions may vary in different countries. In this case, we chose three countries based on their scores in Hofstede (2001)’s individualism and collectivism scale that has been repeatedly validated in cross-cultiral research (Basabe and Ros, 2005) and is widely used in educational, environmental, social, and organizational research (Moon and Sánchez-Rodríguez, 2021; Bruno et al., 2023; Helferich et al., 2023; Kramer, 2023). Following this scale, we selected United Kingdom as a highly individualistic country, with a score of 20. China as a highly collectivistic country, with a score of 89, and Spain as country that stands in between, with a score of 51.

Using behaviors that are considered equally uncivil in the three countries, we expect participants of the United Kingdom, Spain, and China to consider the agent as equally uncivil given that they will be presented as performing equally uncivil behaviors in the three countries (Hypothesis 1a). Whereas we expect their perception of immorality to increase the more collective their culture is, for example, participants from China will consider the transgressor as more immoral than participants from the United Kingdom (Hypothesis 1b).

While some studies suggest that immoral behavior contributes to increased cultural differences in causal attribution (Miller, 1984), with Westerners more inclined to emphasize internal dispositional factors such as personality traits, and Easterners more likely to attribute behavior to external situational factors (Morris and Peng, 1994; Choi and Nisbett, 1998; Masuda and Kitayama, 2004), it might be anticipated that Westerners are more prone to perceive individuals as immoral after witnessing them engage in such behavior compared to Easterners. This assumption may not hold true for transgressions related to social norms of civility. However, research has demonstrated an asymmetry in the perception of (im)moral and (un)civil behaviors (Rodríguez-Pérez et al., 2022). Immoral behaviors are often considered more objectively identifiable than moral behaviors, while civil behaviors are deemed more objectively identifiable than uncivil ones. This implies that recognizing what is immoral is easier than recognizing what is uncivil. Furthermore, there exist disparities in behaviors classified as immoral or uncivil between Eastern and Western countries (Buchtel et al. (2015)). While a substantial number of behaviors are universally categorized as uncivil, Eastern countries tend to identify a higher number of behaviors as uncivil, and these behaviors are often perceived as immoral as well. This phenomenon is less pronounced in Western countries. In a way, this observation aligns with prior research highlighting Easterners’ strong emphasis on contextual factors and their dedication to preserving social harmony (Son, 2012). Given these insights, it is plausible to expect that, in cases involving incivilities or breaches of social norms, Easterners would be the ones to exhibit a greater tendency to be more critical of individuals who commit such transgressions. This is consistent with the distinctive self-concept prevalent among non-WEIRD Easterners, which is shaped by the significance of social relationships (Markus and Kitayama, 1991).

Following the previous reasoning, we also expect differences in the extent to which they feel discomfort in these situations (Moon et al., 2018). Specifically, we expect participants to feel more discomfort when they are from highly collectivistic cultures (Hypothesis 1c). Additionally, we expect differences in their reactions of social control (Moisuc et al., 2018) where it has been observed in Western countries that the more collectivistic is a country, the more they will enact social control (Brauer and Chaurand, 2010). And finally dehumanization (Chen-Xia et al., 2022) toward the transgressor, that is, we expect participants from collectivistic cultures the ones to enact more social control (Hypothesis 1d) and dehumanize more (Hypothesis 1e). Finally, we expect a mediating effect of discomfort on the immorality of the agent based on culture; that is, we expect that the participants of more collectivistic countries experience more discomfort when facing these uncivil behaviors which is what lead them to consider the transgressor as an immoral person (Hypothesis 2).

## 2. Method

### 2.1. Participants and design

The sample (*N* = 398) consisted of 131 British people from the general population who identified as British (63 women, *M*_age_ = 34.64 years, *SD*_age_ = 9.02), 144 Spanish people from the general population who identified as Spanish (73 women, *M*_age_ = 28.47 years, *SD*_age_ = 8.36), and 123 Chinese people from the general population who identified as Chinese (66 women, *M*_age_ = 29.50 years, *SD*_age_ = 6.91). All participants gave their informed consent and received financial incentives in exchange for their participation.

The study followed a single factor between-subjects design, with the independent variable being the culture with three levels based on the nationality of the participants (British vs. Spanish vs. Chinese). All participants received a questionnaire in their native language where they were presented with a person performing three uncivil behaviors. Questions measured six dependent variables for each behavior: behavior incivility, agent incivility, agent immorality, discomfort, social control, and dehumanization. G*Power 3.1 (Faul et al., 2007) suggested we needed 390 participants to detect a medium effect size (*f* = 0.20) with 95% power (*α* = 0.05).

### 2.2. Materials

#### 2.2.1. Type of behavior and background story

We selected 3 uncivil behaviors from Rodríguez-Gómez et al. (2022)’s database (“damaging the street furniture,” “throwing papers and garbage in the street,” and “jumping the queue”) and conducted a pretest (*N* = 77) where participants rated to what extent they considered the behavior uncivil (“Taking into account that Civility refers to behaviors related to courtesy and respect for others. Please indicate on a scale from 1 *not at all uncivil* to 7 *extremely uncivil* to what extent you believe that such behavior is uncivil”) and negative (“Please indicate on a scale from 1 *not at all inappropriate* to 7 *extremely inappropriate* to what extent you believe this behavior is inappropriate”). The three behaviors presented were framed in this context: “Imagine that the following happens to you: you are outside your house on the street when you suddenly see a person who is (damaging the street furniture/throwing papers and garbage on the street/jumping a queue)”.

Analysis of the responses showed that the three behaviors were perceived significantly as uncivil when compared to the midpoint of the civil scale (4.0). Specifically, “damaging street furniture” had a mean of 6.3, *SD* = 1.17, *t*(76) = 17.23; *p* < 0.001, *d* = 1.96, 95% CI [1.58,2.35]; “throwing papers and garbage in the street” had a mean of 5.96, *SD* = 1.44, *t*(76) = 11.98; *p* < 0.001, *d* = 1.36, 95% CI [1.05, 1.67], and “jumping the queue” had a mean of 5.26, *SD* = 1.56, *t*(76) = 7.09; *p* < 0.001, *d* = 0.81, 95% CI [0.55, 1.06]. Likewise, the three behaviors were significantly negative. Specifically, “damaging street furniture” had a mean of 6.13, *SD* = 1.22, *t*(76) = 15.35; *p* < 0.001, *d* = 1.75, 95% CI [1.39,2.10]; “throwing papers and garbage in the street” had a mean of 6.12, *SD* = 1.32, *t*(76) = 14.10; *p* < 0.001, *d* = 1.61, 95%, CI [1.26, 1.94], and “jumping the queue” had a mean of 5.08, *SD* = 1.68, *t*(76) = 5.64; *p* < 0.001, *d* = 0.64, 95% CI [0.40, 0.89].

#### 2.2.2. Behavior incivility (as control)

We adapted an item from the incivilities study by Chen-Xia et al. (2022) to measure the incivility of the behavior. Specifically, participants rated the civility of the behavior on a 7-point scale with endpoints labeled 1 = *not at all uncivil* and 7 = *extremely uncivil*. We expected Chinese, Spanish, and British participants to rate the uncivil behavior as equally uncivil.

#### 2.2.3. Agent incivility

We adapted an item from the study of incivilities by Chen-Xia et al. (2022) to measure the incivility of the agent. Specifically, participants rated the civility of the agent on a 7-point scale with endpoints labeled 1 = *not at all uncivil* and 7 = *extremely uncivil*. We expected all participants to rate the uncivil agent as equally uncivil.

#### 2.2.4. Agent immorality

Participants also rated the immorality of the agent on a 7-point scale with endpoints labeled 1 = *not at all immoral* and 7 = *extremely immoral*. We expected Chinese participants to rate the agent of uncivil behaviors as more immoral than Spanish participants, and Spanish participants more than British participants.

#### 2.2.5. Discomfort

We adapted an item from the study of Moon et al. (2018) to measure how uncomfortable it was to see a person performing that behavior. Specifically, participants rated their discomfort on a 7-point scale with endpoints labeled 1 = *not at all uncomfortable* and 7 = *very uncomfortable*. We expected higher ratings of discomfort from Chinese participants than Spanish participants, and higher ratings from Spanish participants than British participants for uncivil situations.

#### 2.2.6. Social control

We adapted an item from the study of incivilities by Brauer and Chaurand (2010) to measure social control. Specifically, participants indicated to what extent they would react to that behavior, expressing disapproval to the agent, on a 9-point scale with endpoints labeled 1 = *not at all* and 9 = *very much*. We expected Chinese participants to indicate higher social control than Spanish participants, and Spanish participants higher than British participants for uncivil agents.

#### 2.2.7. Dehumanization

We adapted the Ascent of Human measure of blatant dehumanization by Kteily et al. (2015) to measure dehumanization. Participants were presented with a brief text (“Some people seem highly evolved, while others do not appear to be different from lower animals. Using the image below as a guide, use the sliders to indicate how evolved you consider the person who performs the behavior to be”) and indicated their answer on a 0–100 slide with endpoints labeled 0 = *Least evolved* and 100 = *Most evolved*. We expected Chinese participants to dehumanize the uncivil agent more than Spanish participants, and Spanish participants more than British participants.

#### 2.2.8. Attention check

A true or false item was included at the end of the questionnaire to check their attention (“Please indicate whether the following statement is true or false: One of the questions in the questionnaire was about throwing papers and garbage in the street”). Participants who failed this question would be eliminated from the analysis.

### 2.3. Procedure and data analysis

We collected data using a self-administered online questionnaire on the Qualtrics platform. To do this, we generated an electronic reference for the survey and distributed it to people from the United Kingdom, Spain, and China through the Prolific platform in exchange for economic retribution. Participants were presented with a survey in their native language and were selected based on their nationality, as well as the country where they lived most of their life (Brauer and Chaurand, 2010). Then, the participants were asked to read a situation carefully and imagine that it happens to them. A brief text was presented, adapted for each behavior. For example, “You are outside of your house, on the street, when you suddenly observe a person who is damaging the street furniture.” Each behavior was presented in a random order and six dependent variables were then presented below. In the end, they were asked to answer an attention check item to determine their inclusion in the study.

We used SPSS program 25 version for the analyses. A significance level of 0.05 was set. Descriptive statistics were calculated, and we performed a One-way Analysis of Variance (ANOVA) with culture (British vs. Spanish vs. Chinese) as the independent variable for each dependent variable. Also, the SPSS PROCESS macro (Model 4) developed by Hayes (2018) was used to conduct a mediation analysis. Effects were reported with 95% confidence intervals (CIs), and the bootstrapping method with 5,000 resamples of the data tested the robustness of mediating effects.

## 3. Results

Before conducting the analyses, we carried out a verification of the uncivil behaviors perceived by the participants. To this end, we conducted between-subjects ANOVA (culture: British vs. Spanish vs. Chinese) with behavior incivility as the dependent variable. No significant differences were observed based on the culture of the participants [*F*(2,395) = 0.361; *p* = 0.697; η^2^ = 0.002]. As a result, there were no differences in the evaluation of the incivility of the uncivil behaviors in the three groups (*M* = 6.19, *SD* = 0.66 for British; *M* = 6.23, *SD* = 0.80 for Spanish; *M* = 6.27, *SD* = 0.69 for Chinese). These results show that, regardless of the cultural group, the selected behaviors meet the requirement of being perceived as equally uncivil by participants of the three cultures differing in individualism/collectivism.

### 3.1. Agent incivility and immorality

To determine if the three cultural groups rated the agents as uncivil (H1a) similarly and immoral (H1b) differently, we carried out two separate between-subjects ANOVAs (culture: British vs. Spanish vs. Chinese) with agent incivility and agent immorality as the dependent variables.

**Figure 1:**
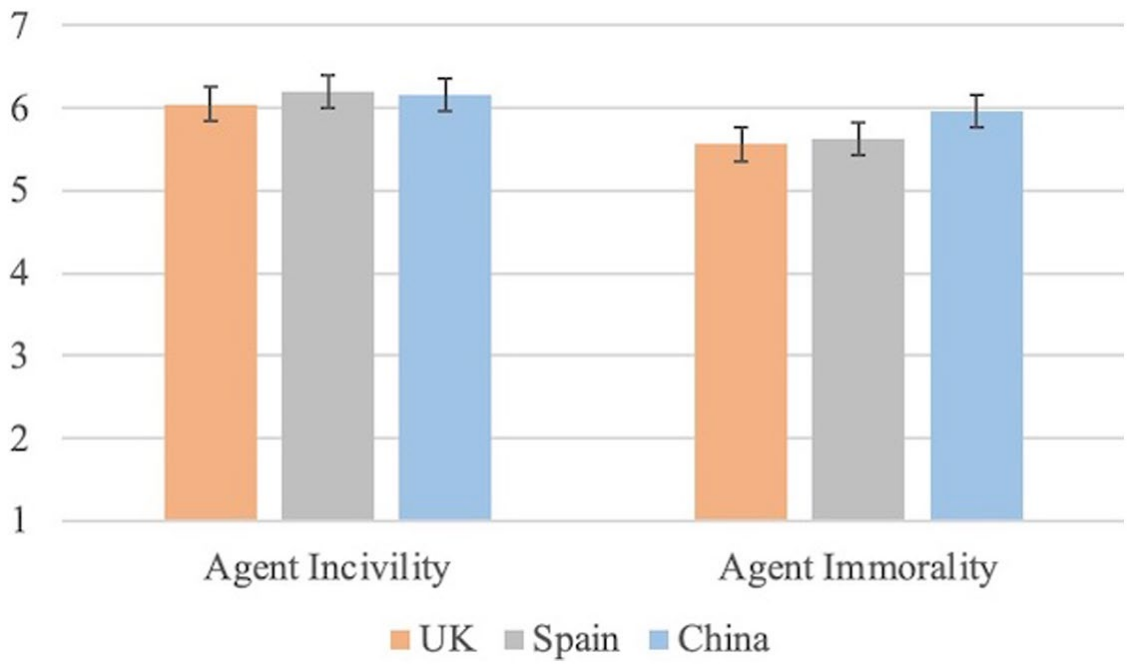
Agent incivility and agent immorality in each country.

As seen in Figure 1, the results related to agent incivility showed no differences based on the culture [*F*(2,395) = 2.395; *p* = 0.093; η^2^ = 0.012; *M* = 6.05, *SD* = 0.75 for British; *M* = 6.20, *SD* = 0.82 for Spaniards and *M* = 6.16, *SD* = 0.76 for Chinese]. However, the ANOVA results showed differences when evaluating the agent immorality [*F*(2,395) = 6.987; *p* = 0.001; η^2^ = 0.034]. Specifically, these differences were between the Chinese participants (*M* = 5.96, *SD* = 0.78) and the British participants (*M* = 5.56, *SD* = 0.88; *t*(252) = 3.76; *p* < 0.001, *d* = 0.481, 95% CI [−0.601, −0.188]) and between the Chinese participants and the Spanish participants (*M* = 5.63, *SD* = 1.00; *t*(265) = 2.96; *p* = 0.003, *d* = 0.368, 95% CI [−0.553, −0.111]). No differences were found between the British participants and the Spanish participants (*t*(273) = 0.544; *p* = 0.587, *d* = 0.074, 95% CI [−0.288, −0.163]). This means that, as expected in H1a, the three cultural groups consider the agent similarly uncivil. Agent incivility is in line with behavior incivility. However, as stated in H1b, Chinese participants rated these equally uncivil agents as more immoral than British and Spanish participants. That is, a person performing a behavior considered uncivil will also be seen as uncivil. However, uncivil agents were also considered immoral by participants of the highly collectivistic culture and not by participants of highly individualistic cultures.

### 3.2. Discomfort

This study is also interested in discovering differences in the experience of discomfort when facing incivility, based on the participants’ culture. The results of the between-subjects ANOVA (culture: British vs. Spanish vs. Chinese) showed significant differences between the cultural groups [*F*(2,395) = 25.62; *p* < 0.001; η^2^ = 0.115]. Specifically, Chinese participants (*M* = 6.01, *SD* = 0.89) showed more discomfort than British participants (*M* = 4.99, *SD* = 1.32; *t*(252) = 7.11; *p* < 0.001, *d* = 0.884, 95% CI [−1.293, −0.732]), and Spanish participants (*M* = 5.55, *SD* = 1.12) also showed more discomfort than British participants (*t*(265) = 3.75; *p* < 0.001, *d* = 0.457, 95% CI [−0.844, −0.263]). In addition, differences between Chinese and Spanish participants were also significant (*t*(265) = 3.65; *p* < 0.001, *d* = 0.441, 95% CI [−0.708, −0.212]), confirming hypothesis 1c. Social norm deviance generates discomfort, and this discomfort varies in people from cultures that differ in individualism/collectivism, with participants from collectivist cultures more affected and experiencing more discomfort.

### 3.3. Social control

To determine to what extent participants considered uncivil behaviors to be socially controlled, a between-subjects ANOVA (culture: British vs. Spanish vs. Chinese) with social control as the dependent variable was conducted. Results showed differences based on the culture [*F*(2,395) = 3.322; *p* = 0.037; η^2^ = 0.017]. Specifically, these differences were between the British participants (*M* = 5.65, *SD* = 1.89) and the Chinese participants (*M* = 6.11, *SD* = 1.71; *t*(252) = −2.01; *p* = 0.04, *d* = 0.255, 95% CI [−0.227, −0.906]) and between the British participants and the Spanish participants (*M* = 6.15, *SD* = 1.65; *t*(273) = −2.33; *p* = 0.021, *d* = 0.282, 95% CI [−0.917, −0.077]). No differences were found between the Chinese participants and the Spanish participants (*t*(265) = 0.182; *p* = 0.856, *d* = 0.024, 95% CI [−0.368, −0.442]). Collectivist cultures enact more social control on those who perform behaviors that transgress social norms. Therefore, the H1d hypothesis is partially accepted because not only Chinese participants but also Spanish participants estimated that they will socially control uncivil agents to a greater extent than British participants.

### 3.4. Dehumanization of the transgressor

The between-subjects ANOVA (one-way) of the culture (British vs. Spanish vs. Chinese) with dehumanization as the dependent variable showed differences between the three cultural groups [*F*(2,395) = 8.38; *p* < 0.001; η^2^ = 0.041]. Specifically, these differences were between the Spanish participants (*M* = 39.61 years, *SD* = 29.48) and the Chinese participants (*M* = 50.18 years, *SD* = 25.96; *t*(265) = −3.08; *p* = 0.002, *d* = 0.380, 95% CI [−17.321, −3.826]) and between the Spanish participants and the British participants (*M* = 52.37 years, *SD* = 27.37; *t*(273) = 3.71; *p* < 0.001, *d* = 0.449, 95% CI [5.990, 19.536]). No differences were found between the Chinese participants and the British participants (*t*(252) = 0.653; *p* = 0.514, *d* = 0.082, 95% CI [−4.411, 8.791]). These results do not confirm H1e, in which we expected a higher tendency to dehumanize transgressors in collectivistic cultures.

### 3.5. Mediation effect of discomfort on the immorality of the agent based on culture

To perform the mediation analysis we used Model 4 in SPSS PROCESS macro (Hayes, 2018) to test the mediation effect of discomfort on the immorality of the agent based on culture. To do that we set the independent variable as categorical and dummy coded with indicator, setting the groups as 1 = Spanish, 2 = British, and 3 = Chinese, to compare theparticipants of the most collective country (China), and the most individualistic country (UK), with the country in between them (Spain).

**Figure 2:**
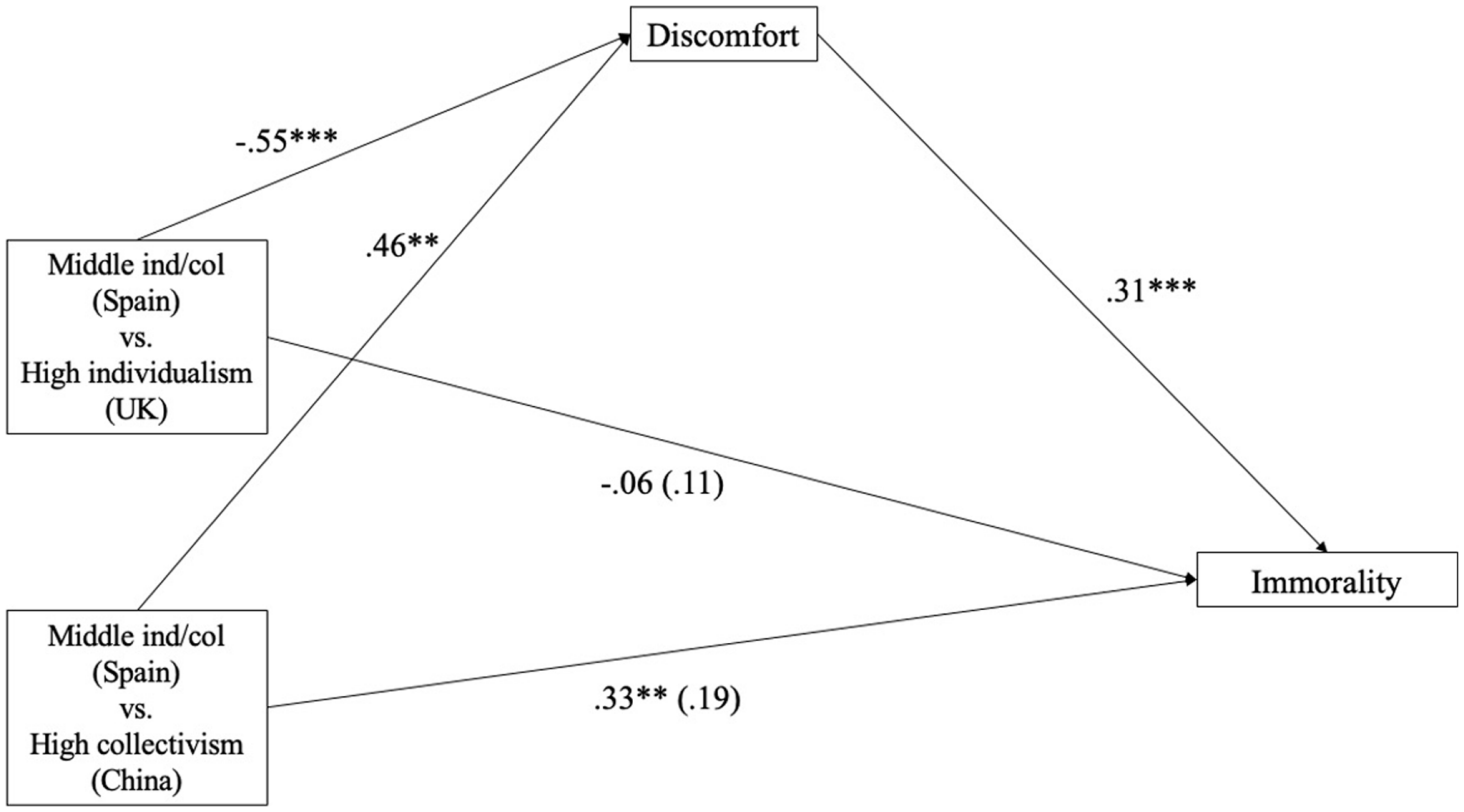
Mediation Effect of discomfort on the immorality of the agent based on culture. *p* < 0.05; **p < 0.01; ***p < 0.001; ind/col, individualism/collectivism.

As seen in Figure 2, the mediation was significant. Culture was associated with discomfort in both cases, when comparing a country that scores in the middle part of Hofstede (2001)’s individualism and collectivism scale (Spain) with a highly individualistic country (UK) (a_1_ = −0.55, *p* < 0.001), the association was negative, whereas when it was compared with a highly collectivistic country (China) (a_2_ = 0.46, *p* = 0.001), the association was positive.

On the other hand, discomfort was positively associated with the immorality given to the agent (*b* = 0.31, *p* < 0.001); meanwhile, the direct effect of culture on the immorality of the agent was not significant in both comparisons (c_1_′ = 0.11, *p* = 0.282; c_2_′ = 0.19, *p* = 0.070), whereas the total effect of culture on immorality of the agent was not significant for Spain vs. UK (*c_1_* = −0.06, *p* = 0.567) but significant for Spain vs. China (*c_2_* = 0.33, *p* = 0.003).

To test the indirect effects, we inspected the bootstrapped CIs with 5,000 samples. The indirect effect was significant in both comparisons. Specifically, culture indirectly affected the immorality of the agent through the mediating pathway of discomfort, decreasing the immorality when it when it was compared with a more individualistic country (*B_1_* = −0.17, SE = 0.05, 95% CI [−0.27, −0.08]), and increasing it when compared with a more collectivistic country (*B_2_* = 0.14, SE = 0.04, 95% CI [0.06, 0.23]).

This is in line with Hypothesis 2. These results show that when people face incivilities, they will face discomfort, and this discomfort increases when they are from more collective countries. Additionally, the more discomfort they feel, the more they will perceive the uncivil transgressor as someone immoral. The individualism or collectivism of a country is not what directly lead people to consider someone who behaved uncivilly as an immoral person, it is the increasing discomfort they feel when facing these incivilities what leads them to perceive the perpetrator as an immoral person.

## 4. Discussion

The aim of the present study was to explore differences between countries in their reaction to social norms transgressions regarding civility based on the cultural dimension of individualism/collectivism. Specifically, we investigated the perception of incivilities and immorality, as well as the experience of discomfort, dehumanization, and social control over the perpetrator of uncivil behaviors varying based on the culture of participants from the United Kingdom, Spain, and China.

The results showed that certain behaviors can be considered equally uncivil in different countries, and all participants perceive the agent of these behaviors as equally uncivil. However, people from highly collectivistic cultures (China) perceive uncivil agents as more immoral than participants from less collectivistic cultures (Spain and the United Kingdom), which is in line with Hypothesis 1a and 1b. These results can be discussed with previous research where what is considered “immoral” or “uncivil” have been studied in different cultures, yet exact behaviors under each definition and overlap were not mentioned nor differentiated. In this sense, Buchtel et al. (2015) observed that when evaluating behaviors, those that were considered uncivil were also seen as immoral in Eastern cultures but not in Western cultures. We observe that this pattern also applies to those that transgress these norms. If you throw garbage in the streets and you are seen as an uncivil person by others, and you are also seen as an immoral person by people from Eastern countries.

These cultural differences regarding incivilities are present not only in the perceptions of agents who perform these behaviors but also in how these transgressions affect observers, as those from highly collectivistic cultures experience more discomfort, which is in line with hypothesis 1c. Moon and Sánchez-Rodríguez (2021) observed that although people from countries with high power distance find incivilities more acceptable, they also feel great discomfort, which may be influenced by their cultural tightness regarding the importance of norms. Following this, our results show a clear distinction regarding the degree of discomfort felt by people from the three different countries that vary in their cultural dimensions of individualism and collectivism, the latter having a stronger reaction toward social norm transgressions. Also, even though we did not confirm hypothesis 1e where we expected collectivist cultures to dehumanize more in the three countries, we did observe it in the western countries. Dehumanization research has been mainly carried in Western countries where the results have been vastly replicated, however, it is unknown how it works in Eastern countries (Zhou and Hare, 2022), the only interaction with the East is when evaluating different presented agents that may be from non-WEIRD countries, or with participants that are not born in Western countries but reside there. In this sense, our results show that although dehumanization also happens in Eastern countries, the way it is enacted may not be in the same way as in Western countries, at least in the case of social norms related to civility.

Finally, our results also showed that the increasing discomfort felt but people from highly collective countries when facing incivilities is what mediates the immorality perceived on the people behaving uncivilly (hypothesis 2), which showed that what leads Western people to consider someone who litters as immoral is not simply because their culture focuses on collectivism, the context or social norms, but the high discomfort they feel in those situations what leads to it in contrast with people from Western countries. This finding can also be related to previous research on gender differences in incivilities where female transgressors faced greater moral outrage than male transgressors and this emotional disparity led to different consequences for them (Chen-Xia et al., 2022). This shows that in the case of incivilities, emotional reactions have an important weight, they differ based on gender, and now, culture is also highly related to emotional reactions linked to incivilities.

Moreover, participants also differed in how they reacted toward the transgressor, dehumanizing and enacting social control over them differently, though these differences were only partially in line with hypothesis 1d. The results of the Eastern country are consistent with previous research carried out in Western countries (Brauer and Chaurand, 2010). Though our hypothesis was only partially confirmed, participants from China and Spain enacted more social control than British participants who are from a highly individualistic country. Even though there were no differences between Spain and China, and Spain is technically an individualistic country by Hofstede (2001), it is one of the most collectivist Western countries.

People comply with norms, be they self-expectations or personal norms, due to enforcement or adherence to their values (Schwartz, 1977; Morris et al., 2015). When these norms are transgressed, the perception of the transgressor and the reaction will be related to how important the norm was to the witness. And the importance of a norm is learned through socialization and influenced by the culture in which an observer is raised, leading to clear personal differences.

The results of the study suggest that the culture plays a significant role in shaping individuals’ perceptions of incivility and social norms violations. In this study, participants from China, Spain, and the United Kingdom showed differences in their evaluation of uncivil behaviors and their transgressors by answering various questions related to their perceptions of immorality, discomfort, dehumanization, and social control over the perpetrator. However, these findings should be interpreted with caution since they are based on a single study with limitations. The number of behaviors and countries used, though based on literature (Hofstede, 2001) and controlled statistically, are high in internal validity but limited, and more examples should be tested for a higher ecological validity. Also, individuals’ cultural values can vary within the same cultural setting based on dispositional traits or due to contextual factors (Mendoza-Denton and Mischel, 2007; Leung and Cohen, 2011). Additionally, future research is necessary to observe if these results can be replicated in highly collectivist Western countries where their primary education is not influenced by Confucian teachings. It would also be interesting to determine if “moral cognition” is universally unique from “norm cognition” in general, given the potential for differing categories of social norms in Chinese and English (Sripada and Stich, 2007; Sinnott-Armstrong and Wheatley, 2013).

In conclusion, the study found that the culture, specifically the individualism and collectivism, does affect individuals’ relations with incivility and immorality. Also, people from collectivistic countries are more likely to experience more discomfort and enact social control over uncivil transgressors. Behaving uncivilly leads to dehumanization, but the degree in which a transgressor is dehumanized may differ in Eastern and Western countries. These findings suggest that cultural values, such as collectivism or individualism, can play a crucial role in shaping individuals’ responses to social norm violations and incivility. Future research is needed to replicate and extend these results, as well as to explore other cultural dimensions that may influence individuals’ responses to social norm violations.

## Data availability statement

The datasets presented in this study can be found in online repositories. The names of the repository/repositories and accession number(s) can be found at: https://osf.io/axbpn/?view_only=35e03176b01c440882debf260d5edb18.

## Ethics statement

The studies involving humans were approved by the study was conducted according to the guidelines of the Declaration of Helsinki and approved by the Ethics Committee on Research and Animal Welfare of the University of La Laguna (CEIBA2017- 0266). The studies were conducted in accordance with the local legislation and institutional requirements. The participants provided their written informed consent to participate in this study.

## Author contributions

XC-X contributed to the data curation, formal analysis, investigation, methodology, software, visualization, writing – original draft, writing – review, and editing. VB contributed to the project administration, conceptualization, funding acquisition, supervision, writing – review, and editing. LR-G contributed to the supervision and editing. AR-P contributed to the conceptualization, funding acquisition, investigation, project administration, resources, supervision, writing – original draft, writing – review, and editing. All authors contributed to the article and approved the submitted version.
